# Tensorial elastic properties and stability of interface states associated with Σ5(210) grain boundaries in Ni_3_(Al,Si)

**DOI:** 10.1080/14686996.2017.1312519

**Published:** 2017-05-02

**Authors:** Martin Friák, Monika Všianská, David Holec, Martin Zelený, Mojmír Šob

**Affiliations:** ^a^ Institute of Physics of Materials, Academy of Sciences of the Czech Republic, Brno, Czech Republic.; ^b^ Central European Institute of Technology, CEITEC MU, Masaryk University, Brno, Czech Republic.; ^c^ Department of Physical Metallurgy and Materials Testing, Montanuniversität Leoben, Leoben, Austria.; ^d^ Institute of Materials Science and Engineering, NETME Centre, Faculty of Mechanical Engineering, Brno University of Technology, .; ^e^ Department of Chemistry, Faculty of Science, Masaryk University, Brno, Czech Republic.

**Keywords:** *Ab initio* calculations, elasticity, grain boundaries, segregation

## Abstract

Grain boundaries (GBs) represent one of the most important types of defects in solids and their instability leads to catastrophic failures in materials. Grain boundaries are challenging for theoretical studies because of their distorted atomic structure. Fortunately, quantum-mechanical methods can reliably compute their properties. We calculate and analyze (tensorial) anisotropic elastic properties of periodic approximants of interface states associated with GBs in one of the most important intermetallic compounds for industrial applications, Ni_3_Al, appearing in Ni-based superalloys. Focusing on the Σ5(210) GBs as a case study, we assess the mechanical stability of the corresponding interface states by checking rigorous elasticity-based Born stability criteria. The critical elastic constant is found three-/five-fold softer contributing thus to the reduction of the mechanical stability of Ni_3_Al polycrystals (experiments show their GB-related failure). The tensorial elasto-chemical complexity of interface states associated with the studied GBs exemplifies itself in high sensitivity of elastic constants to the GB composition. As another example we study the impact caused by Si atoms segregating into the atomic layers close to the GB and substituting Al atoms. If wisely exploited, our study paves the way towards solute-controlled design of GB-related interface states with controlled stability and/or tensorial properties.

## Graphical Abstract

**Figure:**
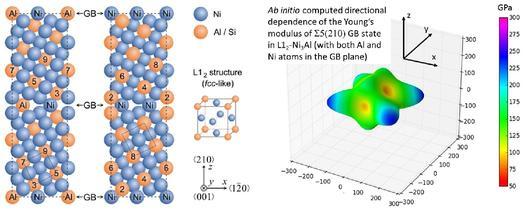

## 1. Introduction

Grain boundaries (GBs) are important extended defects determining a number of material properties. For example, macroscopic strength of polycrystalline materials strongly depends on GB cohesion [1–5]. Quantum-mechanical computational methods are advantageous for theoretical studies of GBs because of their distorted atomic structure [6–20]. Properties of GBs are very sensitive to compositional changes [21–33]. It was found that the impurities even in very low concentrations (ppm) segregated at GBs can drastically change material properties (see e.g. [34,35]). For instance, they can completely suppress local magnetic moments at GBs in magnetic materials as shown by quantum-mechanical calculations for elemental Ni [36,37].

**Figure 1.**
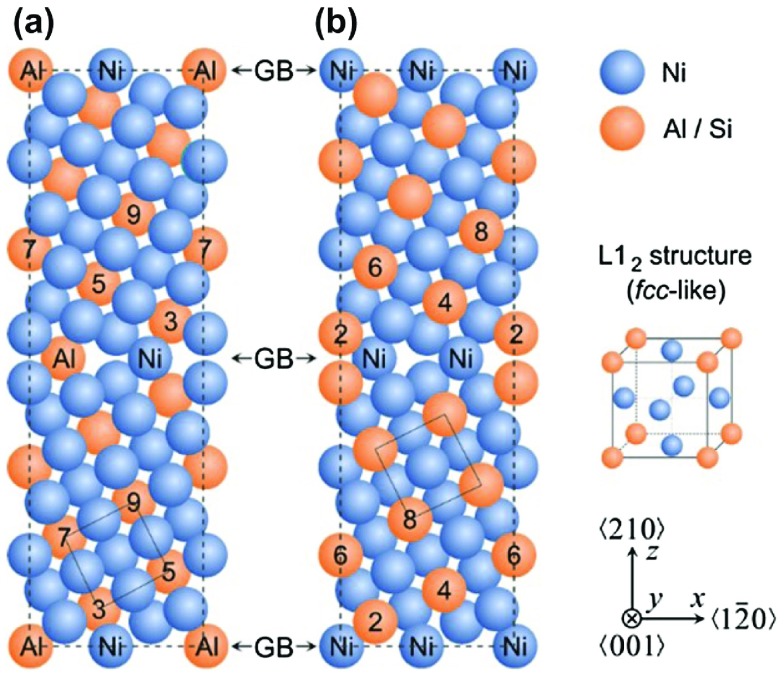
Supercells used in electronic structure calculations of interface states associated with the Σ5(210) GBs in Ni_3_Al with different interface stoichiometries, Σ5(210)^*Al,Ni*^ with both Al and Ni atoms at the GB plane (a) and Σ5(210)^*Ni,Ni*^ with only Ni atoms at the GB plane (b). The blue spheres represent Ni atoms and the orange ones Al (or Si) atoms. The numbers label the atomic layers with respect to the grain boundary (marked by number 1).

Considering the current interest in fabrication, characterization and use of nano-granular materials [38], that can provide unprecedented properties not available in bulk samples, grain boundaries and associated interface states can become even more important than the grain interior. When reaching truly nano-scale grain sizes, even elastic properties (that are typically unaffected in case of larger grains) become dominated by GB states.

The principal goal of our work is to shed more light on tensorial, in particular elastic, properties of materialregions affected by grain boundaries. It is a critically important complement to a number of previous GB-related studies (e.g. [39,40]) that were focused mostly on scalar characteristics (energies, strength, changes in inter-atomic bonds) or electronic and atomic structure (see e.g. [41–54]). The fact that tensorial properties often require much more demanding approaches (see e.g. [55]) is compensated by the wealth of insight that they provide. The knowledge of elastic properties allows for assessing the mechanical stability of the studied system (via generic Born stability criteria [56]). They also play a crucial role in high-temperature thermodynamic stability as they determine long-wave phonon frequencies.

As a case study we selected one of the most important intermetallic compounds for industrial applications, Ni_3_Al, appearing e.g. as the 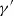 phase in Ni-based superalloys, and the Σ5(210) GB (see Figure 1), which is the smallest one that exhibits an additional volume compared to the bulk. Segregation-driven compositional changes will be exemplified by comparing properties with and without Si atoms substituting Al atoms (Si atoms were experimentally found on Al-sites in Ni_3_Al [57]).

## 2. Methods

Our quantum-mechanical calculations within the framework of density functional theory [58,59] were performed using the Vienna *ab initio* simulation package [60,61]. The exchange and correlation energy was treated in the generalized gradient approximation as parametrized in [62] and implemented in projector augmented wave pseudopotentials [63]. We employed a plane-wave energy cutoff of 500 eV with a 5 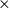 17 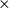 3 Monkhorst-Pack k-point mesh for the 64-atom supercells, reducing the forces on atoms under 1 meV Å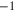. The crystal orbital Hamilton population (COHP) [64] and density of states (DOS) analysis based on projection of plane waves to a local basis [65] implemented in the program LOBSTER [66,67] have been used to understand the interatomic interaction in the studied systems.

The two different GB chemical compositions corresponding to either both Ni and Al or solely Ni atoms at the GB plane are shown in Figure 1(a) and (b). As periodic boundary conditions apply, the supercells are periodic approximants of the real GB-associated interface states. Elastic constants were computed by the stress–strain method outlined in [68,69] and all atomic positions were fully relaxed when simulating the application of external strains. Our tests show that the most critical shear elastic constant, 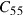, that is as low as 15 GPa in the case when only Ni atoms are located at the interface (see below), would be much higher (64 GPa) if these atomic relaxations are omitted.

**Figure 2.**
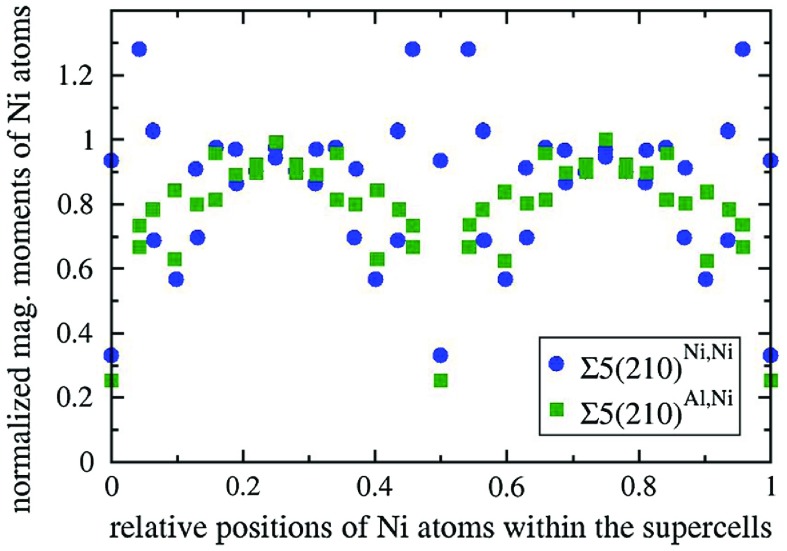
Computed local magnetic moments of Ni atoms normalized with respect to their bulk value as functions of their positions within the computational supercells in the direction perpendicular to the simulated grain boundaries Σ5(210)^*Al,Ni*^ and Σ5(210)^*Ni,Ni*^ in Ni_3_Al. The GBs are located within the supercells at relative positions 0, 0.5 and 1.

As we study a magnetic material, we conveniently use the value of the local magnetic moment to differentiate the GB-affected material from the bulk, but e.g. inter-layer distances in the direction perpendicular to the grain boundary can be alternatively used (see them visualized as a part of the discussion below). Importantly, the size of the supercells is intentionally chosen so that the value of the local magnetic moment of Ni atoms in the bulk Ni_3_Al is reproduced in the 8th atomic layer that is the most distant from the GB interfaces (see Figure 2).

**Figure 3.**
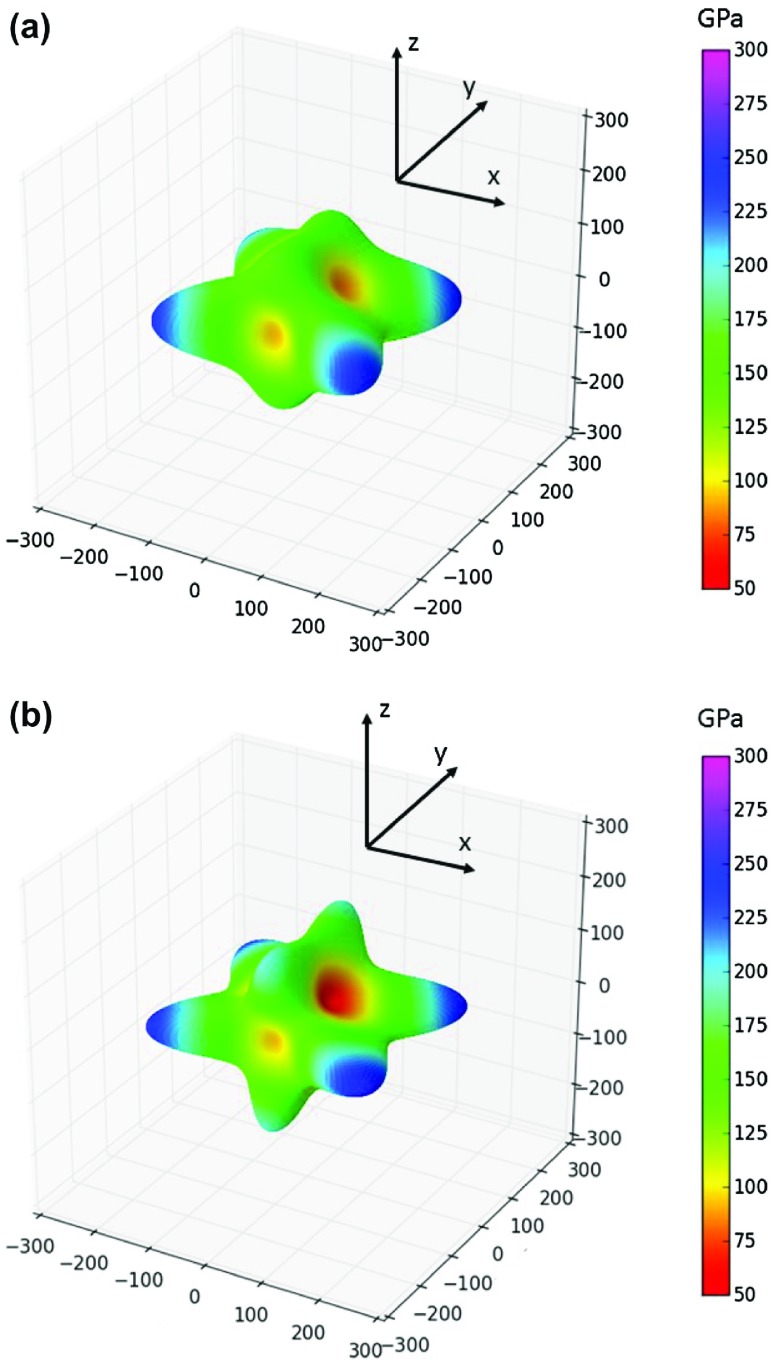
Directional dependences of the Young’s modulus of the studied Σ5(210)^*Al,Ni*^ (a) and Σ5(210)^*Ni,Ni*^ (b) configurations when the GB plane is the *x–y* plane. Displayed dependences were computed and visualized by the SC-EMA [75–77] library (scema.mpie.de) based on quantum-mechanically computed elastic constants (Table 2).

**Table 1.** The values of the two lattice parameters within the (210) plane of studied grain boundaries as obtained by the full relaxation of supercells in comparison with values obtained for the bulk. In particular, the lattice parameters along the *x* 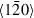 and *y* 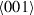 directions (see Figure 1) are listed together with their relative changes with respect to the bulk values.

	Bulk	Σ5(210)^*Al,Ni*^		Σ5(210)^*Ni,Ni*^	
	(Å)	(Å)	(%)	(Å)	(%)
x	7.987	7.920	-0.84	7.917	-0.88
y	3.572	3.583	0.30	3.561	-0.29

In particular, in the case of the Si-segregated configurations, it is only the GB-affected material that is studied. It is an example of the Goldilocks principle when bigger supercells with more atomic layers would contain more bulk-like material, that we do not want to study, while smaller supercells would not contain all the material affected by the extended defect. Tensorial elastic properties of a bulk-like material in bigger supercells would also contribute into the overall elasticity and hinder our effort to determine the properties of the GB-affected material itself. A limiting case of only bulk material being present (and no GB-affected material included) is discussed later in case of an approximative linear-elasticity approach (see below). The supercells have the lattice parameters within the GB plane relaxed, but the values of the two lattice parameters within the (210) plane exhibit only negligible changes when compared with the corresponding bulk values (see Table 1).

## 3. Results

Starting from the bulk Ni_3_Al with the L1_2_ structure, our *ab initio* calculated lattice parameter, 3.571 Å, is in excellent agreement with the experimental value of 3.572 Å  [70]. Regarding Σ5(210) GBs in Ni_3_Al, our calculations show that the Σ5(210)^*Al,Ni*^ with both types of atoms (Al and Ni) at the interface (Figure 1(a)) has a lower GB energy of 1.38 J m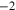 and an additional volume per unit GB area of 0.29 Å  (Å^3^Å^2^) while the Σ5(210)^*Ni,Ni*^ with only Ni atoms at the interface (Figure 1(b)) has a higher GB energy of 1.66 J m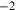 and a larger additional volume per unit GB area of 0.32 Å. The latter values are in excellent agreement with the previous theoretical results of 1.7 J m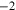 and 0.36 Å  [71].

It should be noted that the additional volume (expressed as a length parameter) is an averaged one when an additional volume obtained for the whole computational cell is divided by the total area of the two GBs inside of the supercell. It differs from vertical atomic-layer-resolved shifts that are analyzed, for example, in the recent study by Kumar et al. [72]. The individual inter-layer distances significantly differ from the bulk equilibrium value (0.799 Å) with the extreme values of these deviations being (i) +0.33 Å for the distance between the GB plane and the first atomic layer off the GB plane and (ii) –0.26 Å between the first and second plane off the GB plane, respectively, in the Σ5(210)^*Al,Ni*^. The extreme values obtained for the Σ5(210)^*Ni,Ni*^ variant of the interface are quite similar, +0.32 Å and –0.20 Å, respectively.

As far as magnetic properties are concerned, Figure 2 shows local magnetic moments of Ni atoms across the computational supercells in the direction perpendicular to the GB interface. In case of Σ5(210)^*Al,Ni*^, all magnetic moments are reduced with the only exception being bulk-like values predicted for the atoms that are the most distant from the GB interface. The reduction of local magnetic moments is found also for a vast majority of atoms in case of Σ5(210)^*Ni,Ni*^ GB variant, but, interestingly, the Ni atoms located in the second and third atomic layer away from the interface have the local magnetic moments increased (by up to 28%). As both GB variants exhibit additional volume with respect to the bulk, a simple magneto-volumetric argument cannot explain significant reduction of most of local magnetic moments within the studied GBs and the clarification will be a subject of future studies.

Focusing on the elastic properties, the three elastic constants computed for cubic Ni_3_Al at T = 0 K are in reasonable agreement with room-temperature experimental data (listed in parentheses [73]): 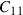 = 243 (224) GPa, 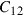 = 152 (149) GPa, 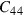 = 128 (123) GPa. Calculated elastic constants of both types of interface states affected by Σ5(210) GBs are given in Table 2 together with bulk elastic constants in the same coordination system. In order to visualize how the interface states associated with GBs respond to uniaxial loading along different crystallographic directions, we exhibit directional dependences of the Young’s modulus in Figure 3.

**Table 2.** The *ab initio* computed elastic constants (all in GPa) of (i) the Ni_3_Al bulk in the coordination system of the studied GBs (shown in Figure 1), (ii) two types of Ni_3_Al#x03A3;5(210) GBs with different atoms (see Figure 1) at the interface, (iii) prediction based on the linear-elasticity approach from [74], and (iv) systems with Si atoms substituting Al atoms in the second layer, Ni_3_(Al,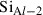) Σ5(210)^*Ni,Ni*^, and eighth layer, Ni_3_(Al,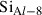) Σ5(210)^*Ni,Ni*^.

Ni_3_Al states									
Bulk 10 001 210	298	152	100	246	152	298	128	76	128
Σ5(210)^*Al,Ni*^	246	152	114	218	148	246	61	29	116
#x03A3;5(210)^*Ni,Ni*^	251	149	105	216	144	244	79	15	118
Linear elasticity [74]	278	152	119	246	152	278	127	71	127
Σ5(210)^*Ni,Ni*^	255	144	128	230	151	238	99	53	107
Σ5(210)^*Ni,Ni*^	256	151	108	219	147	247	79	15	117

The periodic approximants of the GB-related interface states have an orthorhombic symmetry and comparison of Figure 3(a) and (b) for Σ5(210)^*Al,Ni*^ and Σ5(210)^*Ni,Ni*^, respectively, shows how sensitive the elasticity is to compositional changes. The stiffest directions with the Young’s modulus of about 250 GPa (color-coded dark blue in Figure 3) are within the same crystallographic plane as the GB interface while the softest directions with the Young’s modulus approaching only 50 GPa (red colors in Figure 3(b)) are inclined to the GB interface plane.

Analyzing the computed elastic constants listed in Table 2, it is important to note that the obtained shear elastic constants 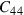 and 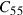 of interface states associated with the Σ5(210) GBs are considerably lower than those in the bulk. The bulk modulus is reduced, too, but only very weakly from 182 GPa (bulk Ni_3_Al) to 171 GPa (Σ5(210)^*Al,Ni*^). In order to rigorously assess these changes and their effect on the mechanical stability, we use Born stability criteria (see e.g. [56]) that connect the mechanical stability with the positiveness of leading principal minors of the matrix of elastic constants. As a consequence, diagonal elements 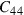, 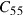 and 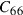 must be positive and the drop predicted for 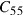 clearly identifies the weakest link.

The interface state associated with the higher-energy GB Σ5(210)^*Ni,Ni*^ has an extremely low value of this crucial elastic constant, 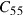 = 15 GPa, i.e. it is less mechanically stable when compared with the interface state related to the lower-energy GB variant Σ5(210)^*Al,Ni*^ with 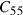 = 29 GPa. This fact is in line with the link between mechanical and thermodynamic stability identified so far only in bulk systems when thermodynamically less stable phases possess also lower mechanical stability (see e.g. [78,79]). Here we show this link between mechanical and thermodynamic stability also in case of interface states associated with GBs.

The predicted reduction of the shear elastic constant 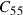 is also in line with previous findings reported for GBs in elemental face-centered cubic metals [80,81]. In particular, results of atomistic simulations were combined with a method [81] that allows decomposition of an overall elasticity into that of different atomic layers and, similarly as in our study, the shear elastic constants were found reduced close to the GB plane. If the same trend of reduction of shear elastic constants is found also for other Ni_3_Al interface regions associated with GBs, it can shed new light on the classical problem related to Ni_3_Al, i.e. its experimentally found very low inter-granular cohesion (see e.g. [82,83]).

## 4. Discussion

Our quantum-mechanical study aims at tensorial properties of interface states associated with grain boundaries, i.e. material formed by only a few atomic layers of atoms close to a particular extended defect. In order to do so, the size of the supercells is carefully chosen to contain only a minimum amount of the bulk material (the grain interior). In particular, the value of the local magnetic moment of Ni atoms in the bulk Ni_3_Al is reproduced only in the eighth atomic layer that is the most distant from the GB planes. It is interesting to address another limit when there is such a high amount of bulk grains that the impact of the interface states related to grain boundaries becomes negligible. In order to do so we apply, in the following, a linear-elasticity approach derived for coherent multilayers (so-called superlattices) by Grimsditch and Nizzoli [74].

**Figure 4.**
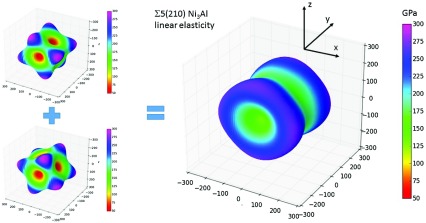
The directional dependence of the Young modulus (in GPa) of the studied interface states associated with grain boundary Σ5(210) as predicted by the linear elasticity theory by Grimsditch and Nizzoli [74] using matrices of *ab initio* calculated elastic constants of bulk Ni_3_Al rotated [84] in the same way as are rotated both grains forming the Σ5(210) grain boundary (the GBs interface is the *x–y* plane).

The linear-elasticity method uses as the input the tensor of elastic constants for bulk Ni_3_Al (see the values in Table 2) that is rotated [84] by two different angles so as to mimic the rotations of the two interfacing grains forming the Σ5(210) grain boundary. The elastic constants obtained in this way correspond to a coherent two-phase multilayer system (stacked along the *z*-coordinate). They are listed in Table 2 and visualized in Figure 4.

The linear-elasticity prediction is different from quantum-mechanical results (Figure 3(a) and (b)) for a number of reasons. First, the linear-elasticity method does not take into account the actual interface properties including its chemical composition or atomic relaxations (only compatibility and equilibrium of normal stress apply). Therefore, Figure 4 presents an approximation that cannot consider any of the two possible elastic characteristics shown in Figure 3(a) and (b) that do depend of the GB interface composition. Second, the linear elastic continuum model is length-scale independent while real atoms and real length dimensions are considered in quantum-mechanical calculations. Third, the input single-crystalline elastic constants were those computed for equilibrium lattice parameter of bulk Ni_3_Al whereas our quantum-mechanically computed elastic constants for interface states related to the Σ5(210) GBs have been determined for fully relaxedgrain-boundary supercells that exhibit additional volume (structural parameters corresponding to the result of the energy minimization).

Importantly, the linear-elasticity approach does not predict any significant reduction of the shear elastic constant 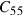. Knowing that the linear-elasticity method, in fact, ignores the actual GB interface with its specific properties including its possible weaknesses, we consider the above comparison as another proof that the softening of 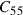 is associated with the studied interface states connected with grain boundaries.

The use of the linear-elasticity method has, in fact, one more motivation. As periodic boundary conditions apply in our calculations, the predicted elasticity is that of a system containing periodically repeated GBs. It would be desirable to extract the elastic constants of a single interface state related to a single GB from our values. Linear-elasticity methods, which use elastic properties of two phases as the input and predict elastic properties of an infinite multilayer system with these two phases coherently co-existing, can be, in principle, inverted in order to meet this target. Unfortunately, the problem seems to be mathematically ill-defined. The number of unknown elastic constants is higher than the number of elastic parameters obtained from our calculations or, in other words, the symmetry of the interface state associated with a single GB is lower than that of its periodic approximant. Therefore, a method (such as in [81,85]), that would determine properties of a single interface state related to a single grain-boundary from results obtained for its periodic approximants remains a challenge for future studies.

**Figure 5.**
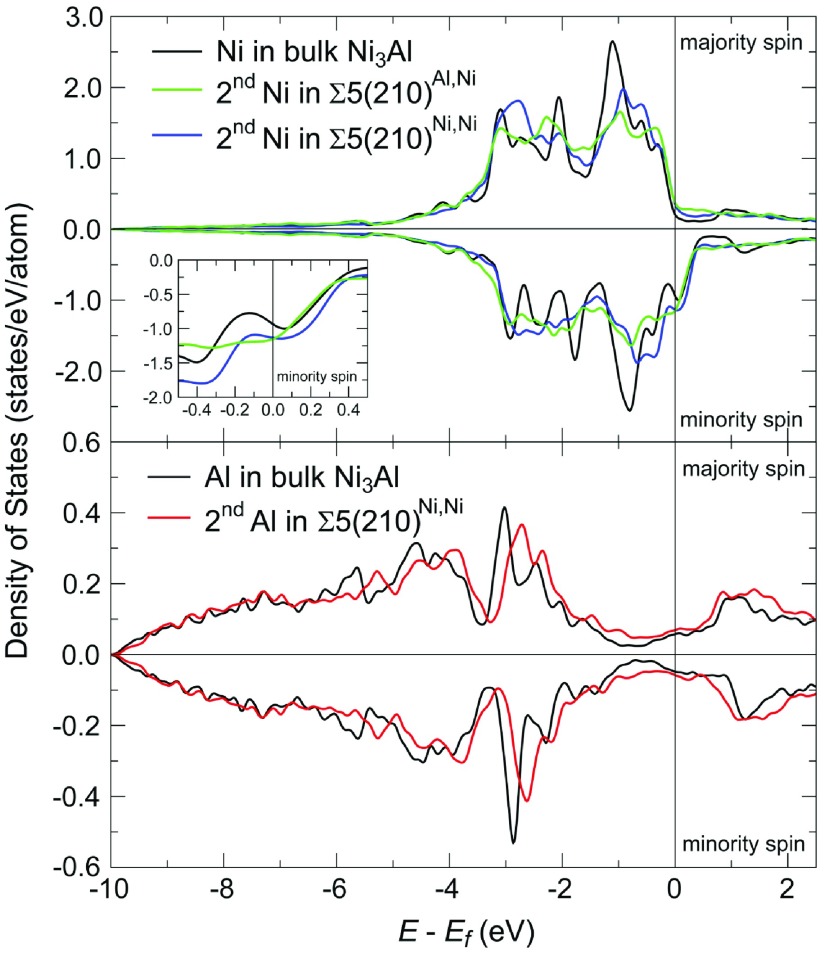
*Ab initio* computed atom- and spin-resolved electronic densities of states for Ni atoms (upper panel) in the bulk Ni_3_Al and in case of Ni atoms from the second atomic layer in both studied Ni_3_Al GBs, Σ5(210)^*Al,Ni*^ and Σ5(210)^*Ni,Ni*^. The lower panel shows the atom- and spin-resolved densities of states of Al atoms in the bulk Ni_3_Al and in the second atomic layer of Σ5(210)^*Ni,Ni*^ GB.

Our quantum-mechanical approach provides a number of other valuable insights. We have employed COHP and DOS analysis to identify the origin of the softening of the 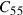 elastic constant discussed above. The atomic resolved DOS of atoms near the grain boundary as well as in bulk Ni_3_Al are shown in Figure 5. The nature of both Ni–Ni and Al–Ni bonds in the bulk material (bond length of 2.53 Å) arises from bonding overlaps of d-d and p-d bands approximately 3 eV below the Fermi level, 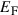 (see the vertical solid black lines in Figure 5). The d-band corresponding to main peak in the DOS of Ni close to 1 eV below the Fermi energy does not participate in bonding. There is also an anti-bonding interaction of Ni d-bands lying just below the Fermi level in the majority spin channel and just above the Fermi energy in the minority spin channel. However, these contributions to bond strength are really small in the bulk material. The bonding behavior of atoms exactly at the grain boundary plane of both studied variants does not significantly differ from behavior of atoms in the bulk.

**Figure 6.**
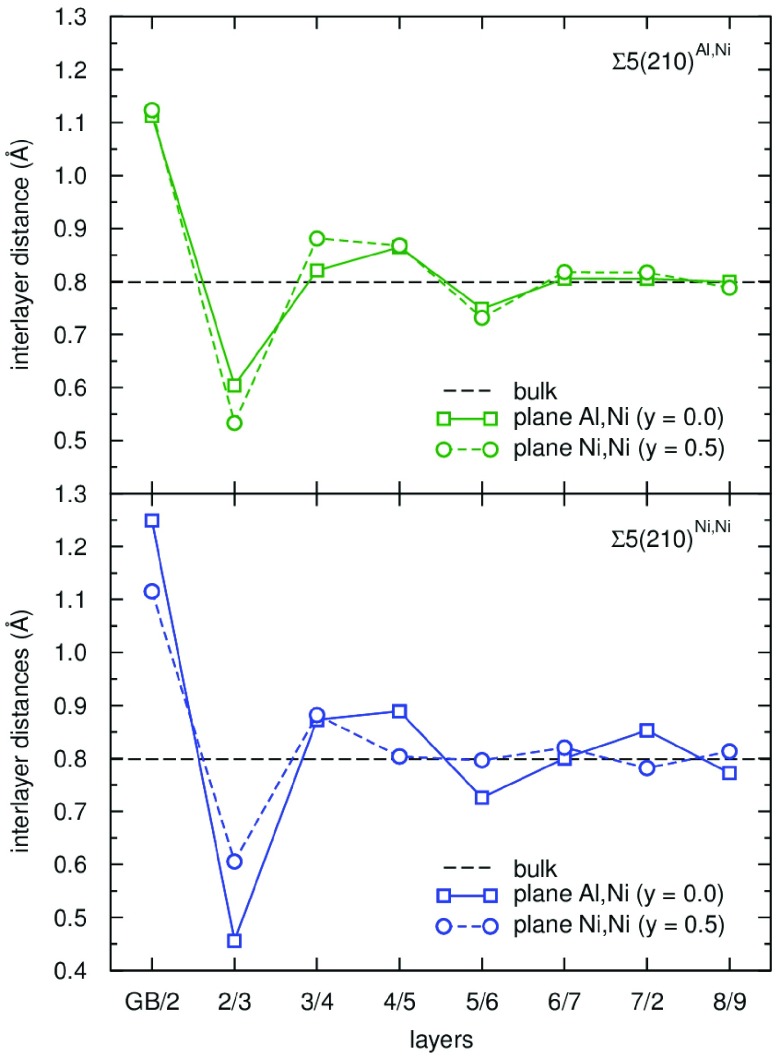
*Ab initio* computed distances between the (210) atomic planes in the supercells modeling Σ5(210)^*Al,Ni*^ and Σ5(210)^*Ni,Ni*^ interface states compared with the value calculated for the bulk.

The origin of the lower mechanical stability can be found in atoms in the second layer and in their mutual interactions across the GB. The Σ5(210)^*Al,Ni*^ contains two Ni–Ni bonds between atoms in the second layer which exhibit the shortest inter-atomic distances (2.23 and 2.25 Å) among all the bonds in the studied systems due to geometry of the GB. Shortening of distances results in a larger overlap of bonding as well as anti-bonding bands and subsequently in higher occupations of anti-bonding states at the Fermi energy in minority spin channel instead of non-bonding states (see green solid line), which destabilizes the whole system. A similar effect can be seen also for Ni–Ni bond across the GB in Σ5(210)^*Ni,Ni*^ with inter-atomic distance equal to 2.23 Å (see solid blue line). The second bond across the boundary plane in this GB variant is between two Al atoms and is significantly longer (2.50 Å), because both Al atoms are strongly bonded to the Ni atoms in the third layer. The Al–Al interaction is weaker than interactions of Ni–Ni atoms across the boundary plane. The weaker Al–Al bond across the GB further destabilizes the whole system compared with the Σ5(210)^*Al,Ni*^ case. The DOS of Al atom in the second layer (see solid red line) exhibits a significant shift of p-bands closer to the Fermi energy. Stronger bonds between Al atom in the second layer and Ni atoms in the third layer can also be recognized from inter-layer distances shown in Figure 6. The value of the distance between the second and third layer (marked as ‘2/3’ in Figure 6) is in the case of atomic planes containing both Al and Ni atoms in Σ5(210)^*Ni,Ni*^ GB significantly shorter than all other inter-planar distances between the second and third layers.

**Figure 7.**
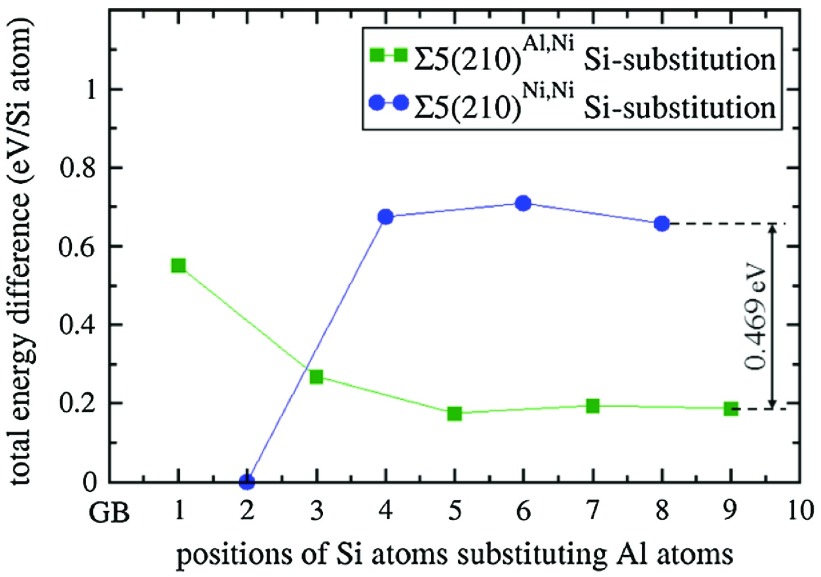
*Ab initio* computed energies of Si-substituted states for both Σ5(210)^*Al,Ni*^ and Σ5(210)^*Ni,Ni*^ GBs expressed as the energy difference with respect to the substituted state with the lowest energy (Ni_3_(Al,Si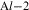) and divided by the number of Si atoms in the supercells (there were two of them). The lines are added only to guide the eye.

Further, to assess elasto-chemical aspects of interface states related to the studied GBs (and their potential for future solute-controlled design of GBs with specific tensorial properties) we further analyze the sensitivity of the elasticity to the chemical composition of the GB plane. We explore the segregation of Si atoms substituting Al atoms and associated elasticity changes. Testing nine different Al positions for Si substituents, the lowest energy was achieved when the Si atom is located at the Al position immediately next to the GB plane, Ni_3_(Al,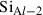), the position marked ‘2’ in Figure 1(b), in the case of the Σ5(210)^*Ni,Ni*^ grain boundary that has only Ni atoms at the GB plane (see Figure 7). The energy favors the Si atoms at position 2 over position 8 by 0.65 eV per Si atom, indicating strong segregation tendencies. The segregation favors the Σ5(210)^*Ni,Ni*^ variant (with the Si substituting an Al atom at position 2, i.e. Ni_3_(Al,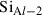)) over the Σ5(210)^*Al,Ni*^ GB variant (with the Si atom substituting Al atom in any of the considered positions in the Σ5(210)^*Al,Ni*^ supercell). It is worth discussing also the energy difference between two close-to-the-bulk states with Si atoms far away from the GB substituting Al atoms at positions 8 and 9, which is marked in Figure 7 and is equal to 0.469 eV per one Si substituent. This energy difference translates into the energy difference of 0.938 eV per whole 64-atom computational cell (with two Si substituents) and this value is very close to the energy difference of 0.962 eV between the supercells modeling Si-free Σ5(210)^*Al,Ni*^ and Σ5(210)^*Ni,Ni*^ GBs.

As the Si segregation alters the thermodynamic preference for the Σ5(210) GB composition variants in Ni_3_Al (Σ5(210)^*Al,Ni*^ variant is preferred without Si atoms while Σ5(210)^*Ni,Ni*^ variant is preferred when Si atoms are nearby), a solute-induced GB phase transformation may occur here.

**Figure 8.**
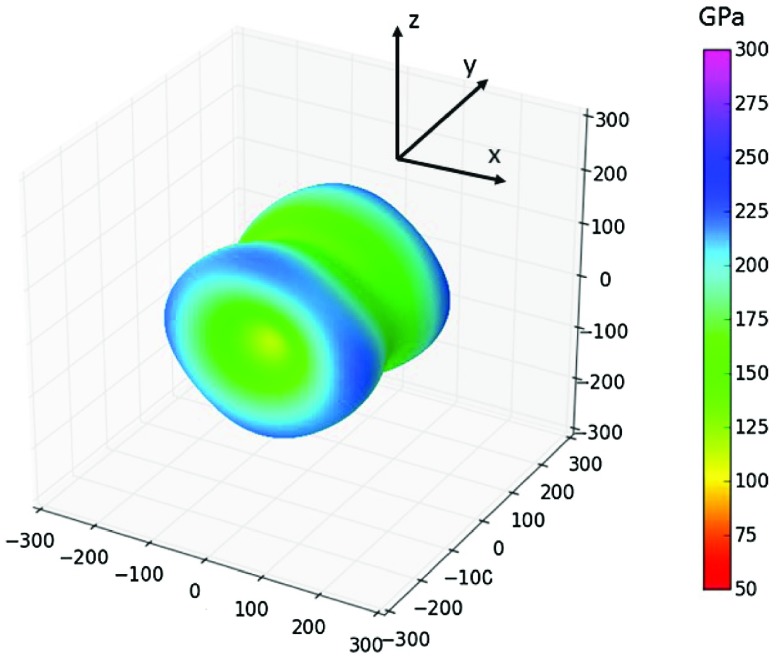
The directional dependence of the Young’s modulus of the studied interface states in Ni_3_(Al, 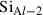) affected by Σ5(210)^*Ni,Ni*^.

The elastic constants of the substituted Ni_3_(Al,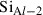) interface state close to the Σ5(210)^*Ni,Ni*^ GB are given in Table 2 and the corresponding directional dependence of the Young’s modulus isvisualized in Figure 8. In contrast to studied Si-free cases, the elastic response is nearly axially symmetric around the *y* axis, the 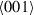 direction, reducing thus the directional anisotropy of elastic properties. Rather accidentally, the directional dependence at Figure 8 shares certain similar features with the linear-elasticity prediction visualized for Ni_3_ Σ5(210) in Figure 4. Importantly, the simulated presence of Si atoms strongly reduces the softening of the shear elastic constant 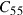 that was discussed above in the case of the interface states associated with the Ni_3_AlΣ5(210) GBs. In particular, the replacement of an Al atom in the second layer by a Si atom completely changes the character of bonding across the GB. The Si–Al bond is stronger than the Al–Al bond and it is able to stabilize the grain boundary. If Al atoms are replaced by Si atoms in the bulk region instead of the GB region, the weak Al–Al bond still exists and the stability of Σ5(210)^*Ni,Ni*^ GB is lower as in the case without Si. Therefore, the Si substitution (leading to the elimination of a weakest link) represents a neat example of options offered by future materials-design of GB interface states.

Our calculations further show that the impact of segregated Si atoms on the elasticity of GB-affected interface states is limited to a few atomic layers close to the GB interface. Putting Si atom at the Al position in the eighth layer, Ni_3_(Al,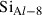) Σ5(210)^*Ni,Ni*^, that has bulk-like properties, provides nearly identical elastic characteristics as the Si-free Σ5(210)^*Ni,Ni*^ (compare the values in Table 2).

## 5. Conclusions

We have applied first-principles calculations to study tensorial elastic properties of periodic approximants of the interface states in Ni_3_Al associated with the Σ5(210) grain boundary. The tensorial elasto-chemical aspects were illustrated by behavior of elastic constants computed for GB-related interface states with different atoms at the GB plane and we find 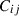 to be strongly sensitive to the GB-plane chemical composition. A three-/five-fold reduction of the elastic constant 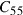 is identified as the crucial weakest link for the mechanical stability. Moreover, when comparing two GB-plane composition variants, we got compositional trends of 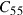 that indicate correlations between the mechanical and thermodynamic stability found previously in bulk systems. Our study thus clearly shows the importance of elastic-constant analysis for next studies of interface states close to GBs when determining their mechanical (in)stability. These should be ideally extended by phonon spectrum calculations, in-plane translation analysis (see e.g. [86]) and simulations of larger deformations (see e.g. [87–92]) in future. The sensitivity of tensorial elasto-chemical properties was also illustrated by studying the impact caused by Si atoms substituting Al atoms in the atomic layers close to the GBs. Nevertheless, the influence of Si atoms at the eighth layer from the Σ5(210) GB in the Ni_3_Al is already negligible. A way towards a solute-controlled design of GB-associated interface states with controlled tensorial elastic properties and stability is thus paved. Finally, considering qualitative links between different tensorial properties postulated by the Neumann’s principle, our study aims at initiating future research addressing tensorial aspects of GB-related interface states.
